# Three dimensional evaluation of the skeletal and temporomandibular joint changes following stabilization splint therapy in patients with temporomandibular joint disorders and mandibular deviation: a retrospective study

**DOI:** 10.1186/s12903-023-02720-w

**Published:** 2023-01-13

**Authors:** Madiha Mohammed Saleh Ahmed, Danli Shi, Majedh Abdo Ali Al-Somairi, Najah Alhashimi, Abeer A. Almashraqi, Mazen Musa, Ning Li, Xi Chen, Maged S. Alhammadi

**Affiliations:** 1 Department of Stomatology, The First Affiliated Hospital of Xi’an Jiaotong University, Xi’an, 710061 Shaanxi People’s Republic of China; 2 Department of Orthodontics, Faculty of Dentistry, Aden University, Aden, Republic of Yemen; 3 Department of Stomatology, The First Hospital of Ningbo, Ningbo, 315000 Zhejiang People’s Republic of China; 4 Department of Orthodontics, School of Stomatology, China Medical University (PRC), Shenyang, 110122 Liaoning People’s Republic of China; 5 Department of Pre-Clinical Oral Health Sciences, College of Dental Medicine, QU Health, Qatar University, Doha, Qatar; 6 Department of Orthodontics, Al Tegana Dental Teaching Hospital, University of Science and Technology, Omdurman, 11111 Khartoum Sudan; 7 Department of Orthodontics, Yantai Stomatological Hospital Affiliated to Binzhou Medical College, Yantai, 264000 People’s Republic of China; 8 Division of Orthodontics and Dentofacial Orthopedics, Department of Preventive Dental Sciences, College of Dentistry, Jazan University, Jazan, Saudi Arabia; 9 Postgraduate Orthodontic Program, Department of Orthodontics, Pedodontics and Preventive Dentistry, Faculty of Dentistry, Sana’a University, Sana’a, Republic of Yemen

**Keywords:** CBCT, Mandibular deviation, Stabilization splint, Temporomandibular disorders, Three-dimensional analysis

## Abstract

**Background:**

Three-dimensional (3D) detailed evaluations of the mandibular mediolateral position, mandibular condylar position, and temporomandibular joint (TMJ) spaces following stabilization splints (SS) therapy in patients with temporomandibular joint disorders (TMD) and mandibular deviation (MD) have not been reported in the available literature. Accordingly, this study aimed to three-dimensionally analyze the skeletal and bony temporomandibular joint changes following stabilization splint therapy in adult patients with temporomandibular joint disorders and mandibular deviation.

**Methods:**

This study is a retrospective clinical study that enrolled 26 adult patients with TMD and MD with a mean age of 24.86 years. The Diagnostic Criteria for Temporomandibular Disorders (DC/TMD) was used to diagnose TMD. SS was adjusted weekly until occlusal contact stabilization occurred, and then adjusted monthly, patients were instructed to wear it at night for at least 10 h. The SS was removed after the elimination of TMD symptoms (TMJ/muscle pain on palpation, muscle spasm, and clicking) and having both condyles completely seated in a musculoskeletally stable position. Pre- and post-therapeutic Cone Beam Computed Tomography (CBCT) was analyzed. Mandibular mediolateral position, TMJ spaces, and mandibular condyle position were analyzed three-dimensionally using Mimics 21.0 software. Paired t-test or Wilcoxon rank-sum test was performed, and the significance level was considered at *P* < 0.05.

**Results:**

The treatment period with SS therapy was 10.07 ± 3.1 months. The deviated chin was improved in 69.23% of the sample; the range of improvement was > 0 mm ≤ 3.9 mm. The mandibular rotation was significantly decreased from 3.58 ± 2.02° to 3.17 ± 1.60. The deviated side’s superior and posterior joint TMJ spaces were significantly increased from 2.49 ± 0.88 mm and 1.25 ± 0.79 mm to 2.98 ± 1.02 mm and 1.86 ± 0.72 mm, respectively. The value of the difference from the bilateral condyle head position to the X and Z axes significantly decreased from 2.50 ± 1.56 mm and 2.30 ± 1.57 mm to 1.64 ± 1.58 mm and 1.82 ± 1.11 mm, respectively.

**Conclusion:**

The main positional effect of the stabilization splint treatment in TMD patients with MD includes considerable correction of mandibular deviation, improving facial asymmetry, and moving the condyle into a stable condylar position; these were done by promoting the mandible to rotate around the Z (roll) and Y (yaw) axes and by forward, downward, and outward condylar movement on the deviated side, respectively.

## Background

Facial symmetry has always been a subject of interest for clinicians, psychologists, and artists. Facial symmetry is an important index for the evaluation of facial beauty. Usually, the human face has limited asymmetry and minimal normal deviation [1]. According to Haraguchi et al. [2], an up to 2 mm of facial midline deviation is indiscernible in a regular face. Facial asymmetry is common in the lower third of the face [1]. In addition, according to Severt et al. [3], 74% of patients with facial asymmetry have chin deviation. Mandibular deviation (MD) showed more frequently on the left than the right side [4]. MD was reported to be frequently occurring in patients with temporomandibular joint disorders (TMD) [5]. In a study conducted by Aileen et al. [6], whose sample was based on an Asian population, it is mentioned that mandibular asymmetry may be a possible etiopathologic factor in TMD because the prevalence of TMD was significantly higher in those with mandibular asymmetry than in those without. Mandibular asymmetry and tilting of the frontal occlusal plane have been hypothesized to cause condylar displacement in the fossa, loading both joints unevenly and ultimately causing TMD. TMDs are a major public health concern that affect about 60%–70% of the general population (at least one TMD sign) [7]. TMD is a collective term that includes several clinical complaints involving the muscles of mastication, the TMJ, and/or associated orofacial structures [8]. The main clinical characteristic of TMD is pain and joint clicking that can be restricted in the temporomandibular joint (TMJ) area or extended to the eyes, shoulder, and neck region [9]. Other common manifestations include fatigue in muscles of mastication, muscle weakness, abnormal mandibular movement, headache, locking, limited mouth opening, and even MD [9].

The management options for TMD include non-surgical and surgical interventions or a combination of both. Approximately 90%–95% of treatment strategies begin with non-surgical treatment, which is considered the most effective management technique for patients with TMD [10], and this treatment includes occlusal splints (OS), counseling therapy, physiotherapy, oral or injectable pharmacotherapy and low-level laser therapy [11]. Approximately 5%–10% of patients with TMD may be suitable for surgical intervention [10], such as arthroscopy and open TMJ surgical procedures. Jung et al. [12] evaluated the effect of orthognathic surgery on temporomandibular joint symptoms. Haraguchi et al. [2] suggested that mandible asymmetry exists when the deviation is more than 2 mm and can be observed when it reaches 4 mm; if the deviation is above 6 mm, an orthognathic treatment is required.

The most non-surgical successful treatment provided for TMD is OS [12, 13]. OS are considered deprogrammers or jaw repositioners to establish ideal maxillomandibular relationships, thus relieving pain and restoring function [14]. The action mechanisms of OS used in treatment include occlusal disengagement, modification of occlusion’s vertical dimension, muscle relaxation, joint unloading, or TMJ repositioning [14, 15]. For the normal functioning of a joint, joint space must be present within the normal range. Movement may be limited if the joint space is diminished; or restricted, which may cause pain. [16]. TMJ osteoarthritis is regarded as present if the joint space narrows or changes. The promotion of SS early therapy may aid in preventing the disease progression [17].

In a recent systematic review, Al-Moraissi et al. [11] concluded that all OS are probably more effective treatments for TMDs compared with no treatment and non-occluding splints. Alqutaibi et al. [14] described the different types of occlusal appliances (flat plane stabilization appliance, anterior bite plane, anterior repositioning appliance, neuromuscular appliances, posterior bite plane, pivot appliances, and hydrostatic appliance), each having its special design indications and precautions that should be followed in TMD management. By contrast, the most widely used types are stabilization splints (SS), anterior repositioning splints, and anterior bite splints [11].

SS can provide centric relation occlusion, eliminate occlusal interferences, offer anterior guidance on anterior teeth, reduce neuromuscular activity, and achieve stable occlusal relationships with uniform tooth contacts across the dental arch and joint stabilization [14, 15, 18]. These functional benefits enable SS to take an active part in the auxiliary diagnosis and treatment of TMD, thus providing a removable and transitory ideal occlusion. So far, three-dimensional (3D) detailed evaluations of the mandibular mediolateral position, mandibular condylar position, and TMJ spaces following SS therapy have not been reported in the available literature. Therefore, this study aimed to three-dimensionally analyze the skeletal and bony temporomandibular joint changes following stabilization splint therapy in adult patients with temporomandibular joint disorders and mandibular deviation.

## Methods

### Patients’ selection

This retrospective clinical study was approved by the ethics committee of Xi'an Jiaotong University, Xi'an Jiaotong University, China (No. XJTU1AF2022LSK-028). Informed consent was obtained from all subjects. Moreover, all methods were carried out in accordance with the principles of the declaration of Helsinki.

### Sample size

The sample size was calculated using G* Power software (Version 3.1.3; Franz Faul, Universität Kiel, Germany) with *α*value of 0.05 and a power of 80% based on a pilot study, in which the changes in the superior and posterior joint spaces of the deviated side were − 0.48 ± 0.86 and − 0.62 ± 1.00 mm, respectively. The resulting sample size was a minimum of 25 or 24 patients. This number was increased to a minimum of 26 patients.

All records of patients diagnosed and treated between July 2017 and July 2019 in the Department of Stomatology, First Affiliated Hospital of Xi'an Jiaotong University, China, were screened. The inclusion criteria included: (1) 18–33 years old; (2) diagnosed with TMD based on the clinical data records following the Diagnostic Criteria for Temporomandibular Disorders (DC/TMD) check list, which includes muscular and/or TMJ pain, TMJ sounds, and the range of the mandibular motions [19], disc derangement with reduction (as clinically diagnosed) and/or myalgia; (3) centric relation/centric occlusion discrepancy of > 1 mm in the vertical plane and > 0.5 mm in the transverse plane [20]; (4) distance from the menton points to the midsagittal plane of ≥ 2 mm due to functional mandibular deviation [2]; (5) normal development of the craniomaxillofacial structures; (6) a full set of permanent dentition except the wisdom teeth; and (7) healthy periodontal condition. The exclusion criteria were: (1) history of craniomaxillofacial trauma; (2) active phase of idiopathic condylar resorption; (3) unilateral condylar hypoplasia or hyperplasia; (4) history of orthodontic, orthognathic treatment, and temporomandibular joint surgery; (5) parafunctional habits and (6) patients with abnormal mental or psychological behavior.

### Stabilization splint fabrication and therapeutic phase

Dental alginate impression material (Jeltrate®, Densply, Dunsberg dental Co. Ltd, Tianjin, China) of the upper and lower arches were obtained, and then dental casts were poured following the manufacturer’s instruction (Die Stone, Kluzer Modern Materials®, Indiana, USA). The Roth power centric technique [21] was used to register the centric relation (CR) immediately following neuromuscular deprogramming with the patient relaxed and reclined at 45°. Later, the CR was recorded by two-piece wax registration consisting of anterior and posterior sections (Delar Bite Registration Wax; Delar® Corporation, Lake Oswego, OR, USA) in the maximum intercuspation (MIC) by a single layer wax record (Moyco2® Industries Inc., Philadelphia, PA, USA). Furthermore, the facebow was established to record the relationship of the upper teeth to the anatomical reference area and transfer this relation to the articulators. Later, dental casts, facebow, and wax records were transferred and mounted on a semi-adjustable articulator (AD 2®; Advanced Dental Designs Inc, Riverside, USA). The horizontal, vertical, and transverse condylar position (CP) were evaluated using a single Measures Condyle Displacement (MCD) device and MIC wax record. The Helkimo Index was used to evaluate the patients’ clinical condition [22].

The dental casts in the articulator had the same opening axis as teeth related to the TMJ. After fabricating the SS, it was inserted into the patient mouth, and occlusal adjustments were performed using a double-sided articulating film (Accufilm II, Parkell Inc., NY, USA) [23]. No polishing procedure was accomplished after adjusting the occlusal splint to preserve the occlusal contacts [23].

SS is a flat, hard acrylic occlusal splint that provides a removable and transitory optimum occlusion. Splint therapy provides an optimal occlusion, decreases excessive muscle activity, and provides neuromuscular equilibrium [24]. The patients were asked to wear the SS at night and for at least 10 h [25]. The SS needs to be adjusted over several visits. After 1 week of SS use, the bite marks on the occlusal surface of the splint were checked and re-adjusted by grinding, and the refined stable jaw position was acquired. The procedure was repeated weekly until occlusal contacts were stabilized, and the patients returned every 2 weeks. The patients were recalled 2 months later, and a follow-up visit was extended and reviewed at regular intervals as appropriate to ensure a mutually protected occlusion [22, 23, 26]. After months of treatment, SS was continued worn until TMD signs and symptoms were eliminated, and the patient reported to be pain-free and having both condyles completely seated in a musculoskeletally stable position.

Thus, following treatment, the Helkimo index and MCD were recorded. When the test showed that the condyles were close to the CR, the TMD symptoms of pain in the masticatory muscles, TMJ, neck and shoulders, and TMJ/muscle pain on palpation, headache, muscle spasm, clicking and popping in opening and/or closing, and deviation in opening and/or closing, the limitation in mouth opening was improved in the three consecutive follow-up visits by using splint therapy only; the patient was instructed to stop wearing the appliance after reaching stable TMJ position and being relieved of the symptoms [22]. Although SS can improve the clinical symptoms of patients with TMD, prolonged used is not recommended [27], because it can cause clockwise rotation of the mandible.

### CBCT assessment

Three-dimensional images were acquired using the CBCT machine (KaVo 3D eXam; KaVo Dental, Bismarckring, Germany). The following CBCT imaging parameters were set: 120 kV, 37.1 mA, field of view of 23 cm × 17 cm, exposure time of 17.8 s, voxel size of 0.3 mm, and a slice thickness of 0.3 mm. The patients were sitting upright with their teeth closed to their maximum intercuspation. The Frankfort horizontal plane was positioned parallel to the floor and the midsagittal plane perpendicular to the floor; all patients were instructed not to swallow during scanning. The collected CBCT scan data were transferred into DICOM (Digital Imaging and Communication in Medicine) file format and then imported into Mimics 21.0 software (Materialise Company, Belgium) for 3D reconstruction.

To ensure that the measurements were properly done on the 3D images, the following were considered: (1) adjustment of the coordinate system in the three planes of space was done prior to any landmark localization to counter for any positioning error during scanning; (2) the landmarks digitization was not done the section module but on the 3D module guided by slice locator (sagittal, axial and coronal) to have more precise 3D localization; and (3) the selected measurements were re-estimated within a 3-week interval by the intra-observer and inter-observer assessment of the whole sample to evaluate the intra- and inter-examiner reliability of the studied measurements.

**Fig. 1:**
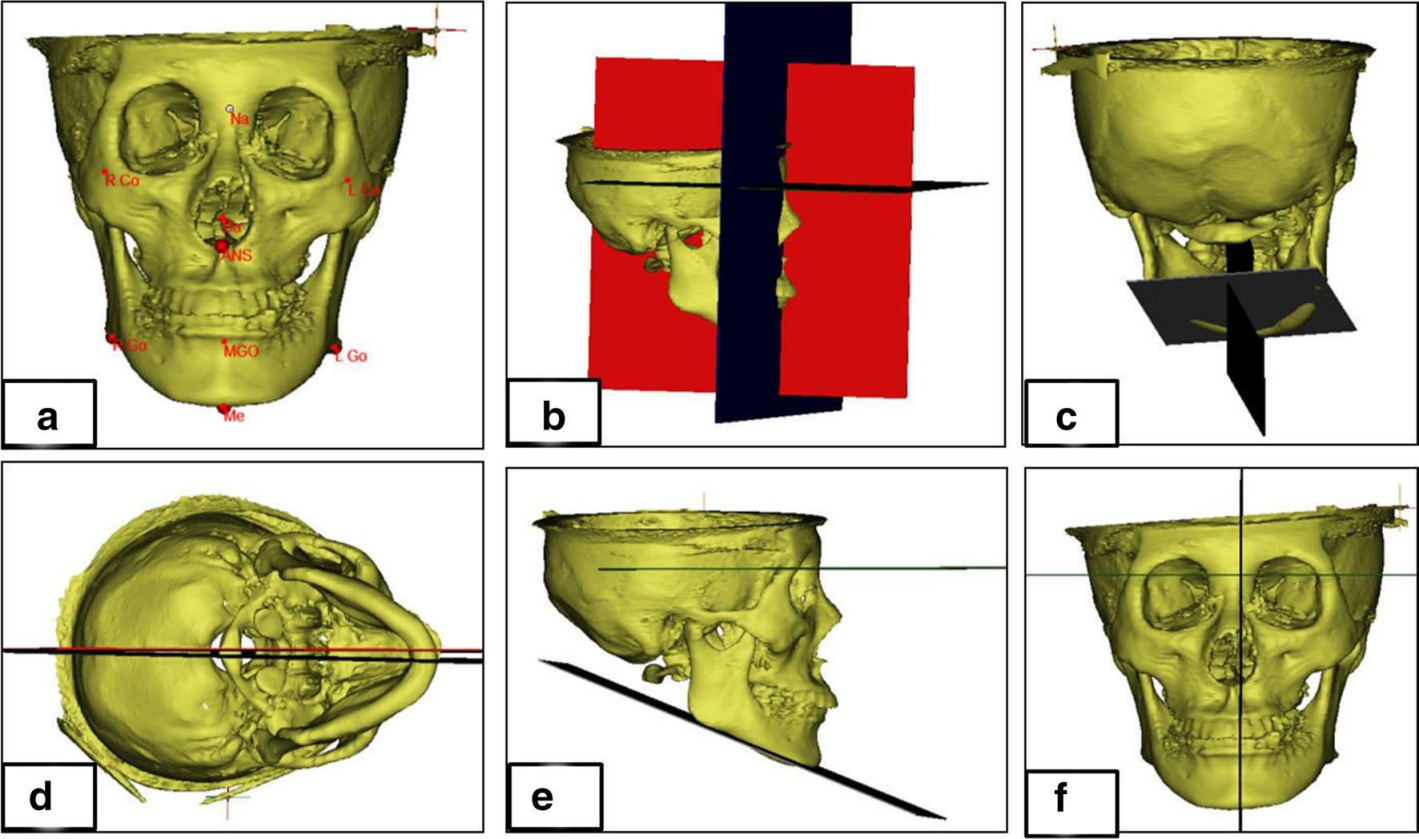
Three-dimensional fixed anatomical landmarks, reference planes, and mandibular skeletal measurements: **a** anatomical skeletal landmarks; **b** 3D coordinate reference planes (red: FMSP, blue: CP, and green: HP); **c** mandibular reference planes (gray: MP and green: MMP); **d** mandibular rotation; **e** mandibular divergence; and **f** mandibular canting

**Fig. 2:**
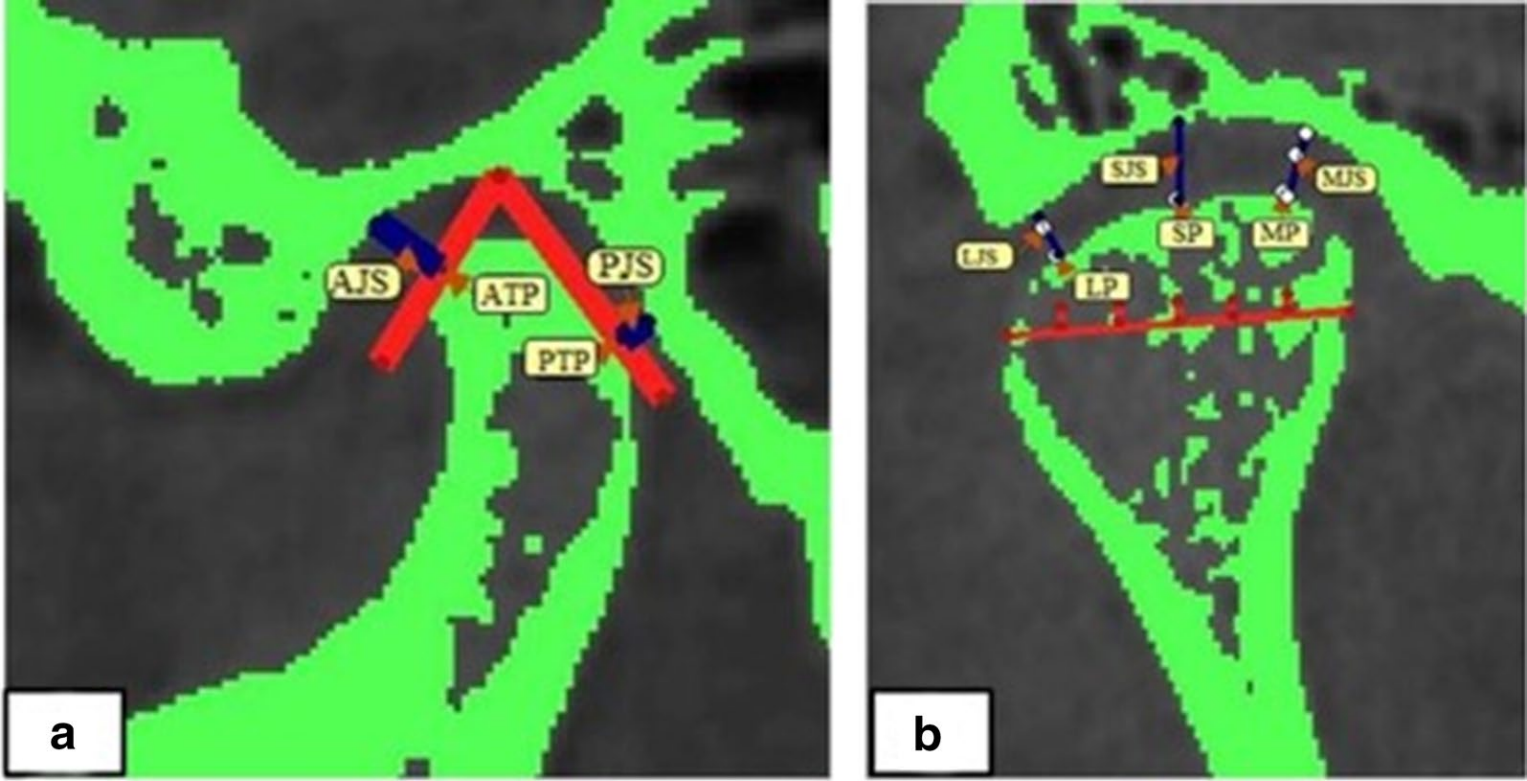
Landmarks and TMJ space measurements **a** Anterior Tangent Point (ATP), Posterior Tangent Point (PTP), Anterior Joint Space (AJS), and Posterior Joint Space (PJS); **b** Superior Point (SP), Medial Point (MP), Lateral Point (LP), Superior Joint Space (SJS), Medial Joint Space (MJS), and Lateral Joint Space (LJS)

According to Haraguchi et al. [2], the chin deviation (CD) analysis was measured from the facial midline to the menton point; it was considered as a deviation of more than 2 mm. The 3D anatomical landmarks, reference planes, TMJ spaces, 3D condylar position [28], and mandibular measurements [29] are presented in Table 1 and Fig. 1. According to methods described by Alhammadi et al. [30, 31], the TMJ spaces (anterior joint space “AJS”, posterior joint space “PJS”, superior joint space “SJS”, medial joint space “MJS”, and lateral joint space “LJS”) were measured in millimeters (Fig. 2).

**Table 1:** Definition of anatomical landmarks, skeletal reference planes and measurements used in the study

Identification	Abbreviation	Definition
Anatomical Landmark		
Nasion	N	Anterior and superior frontonasal suture
Basion	Ba	The foramen magnum’s inferior-anterior margin in the skull base midline
Orbital	Or	The orbit lowest midpoint at its inferior border
Anterior Nasal Spine	ANS	The maxillary anterior nasal spine most anterior point
Gonion	Go	The intersection of the bisecting angle point between the mandibular plane and the mandibular ramus plane at the mandibular angle
Menton	Me	The lowest bony point of the chin at the MD symphysis
Condylion	Co	Most superior point of the condyle
Mid-Gonion	MGo	The midpoint between left and right Gonion
Reference Planes		
Facial midsagittal plane	FMSP	The plane constructed by (N), (BA), and (ANS) passing through (N) as the coordinate origin
Orbital-Facial midsagittal plane	Or-FMSP	A plane passing through right orbital, left orbital and perpendicular to FMSP
Horizontal plane	HP	A plane parallel to Or-FMSP passing through (N) as the coordinate origin
Coronal plane	CP	The plane perpendicular to both (FMSP) and (HP) passing through (N) as the coordinate origin
Mandibular Plane	MP	The plane constructed by left and right (Go) and (Me)
Mid-mandibular plane	MMP	The plane passing through the (MGo) and (Me) and perpendicular to the mandibular plane
Three Dimensional Measurements		
*3D, TMJ joint spaces*		
Anterior Joint Space	AJS	The shortest distance from the fossa to the sagittal anterior tangent point (AC) of the condyle
Posterior Joint space	PJS	The shortest distance from the fossa to the sagittal posterior tangent point (PC) of the condyle
Superior Joint Space	SJS	The shortest vertical distance from the mid-point of the total width on the condyle surface to the opposing fossa wall
Medial Joint Space	MJS	The shortest vertical distance from the junction of the medial first and second sextants of the condyle to the opposing fossa wall
Lateral Joint Space	LJS	The shortest vertical distance from the junction of the lateral first and second sextants of the condyle to the opposing fossa wall
*3D Condyle position*		
Condylion-X axis	Co-x	The distance from Condylion (Co) to midsagittal plane
Condylion-Y axis	Co-y	The distance from Condylion (Co) to horizontal plane
Condylion-Z axis	Co-z	The distance from Condylion (Co) to the coronal plane
*3D Mandible*		
Chin deviation (mm)	CD	The horizontal distance from Me point to FMSP
Mandibular rotation (°)	FMSP-MMP	The intersection angle between FMSP and MMP (Z-axis)
Mandibular canting (°)	HP-MMP	The angle between HP and MMP (Y axis)
Mandibular divergent (°)	MP-HP	The intersection angle formed by MP and HP intersecting at Gn (X-axis)

The TMD symptoms were considered as predictor variables. Mandibular deviation improvement was considered as primary outcome while TMJ spaces, condylar position and mandibular angles were considered as secondary outcomes.

### Statistical analysis

All data were analyzed using Statistical Package for Social Sciences (SPSS) 25.0 software (IBM Corp., Armonk, NY, USA). The normality data were evaluated using Shapiro–Wilk test. Paired t-test or Wilcoxon rank-sum test was performed to compare the differences in CD, TMJ space, condyle, and mandibular position measurements before and after treatment. The Pearson correlation was used to assess the relation between the change in the chin deviation and the joint spaces. The statistical significance level was set at P < 0.05.

## Results

A total of 28 patients with TMD and mandibular deviation aged 18–33 years (average of 24.86 ± 5.04 years) were enrolled. The treatment period with SS therapy was 10.07 ± 3.10 months. A good to excellent intra- and inter-observer reliability was found; with a minimum value of 0.783 for mandibular divergent angle and maximum value of 0.998 for lateral joint space measurements.

According to the comparative analysis of CD for pre- and post-treatment, Table 2 shows the percentage of patients having CD pre-treatment and the improvement of the patients’ chin deviation post-treatment having CD from the distance of Me to FMSP (x) with 2 mm ≤ x < 4 mm, 4 mm ≤ x < 6 mm, and x ≥ 6 mm pre- and post-treatment. According to the total change and improvement of CD post-treatment, 69.23% of patients had decreased CD post-treatment, while 30.77% had an improvement between 0 mm < x < 1 mm, 23.08% had an improvement between 1 mm ≤ x < 2 mm, 7.69% had an improvement between 2 mm ≤ x < 3 mm, and 7.69% had an improvement of x ≥ 3 mm. The range of improvement was 0 < x ≤ 3.9 mm.

**Table 2:** Percentage (%) of improvement in chin deviation

	Chin deviation (%)		
	2 ≤ X < 4 mm	4 ≤ X < 6 mm	X ≥ 6 mm
Pre treatment	50	26.92	23.08
Post treatment	69.23	19.23	11.54

Variation range of Chin deviation improvement (%) post treatment					
	X ≤ 0 mm	0 < X < 1 mm	1 ≤ X < 2 mm	2 ≤ X < 3 mm	X ≥ 3 mm
Post treatment	30.77	30.77	23.08	7.69	7.69

The measurement results of TMJ spaces (AJS, PJS, SJS, MJS, and LJS) before treatment with TMD and MD show that the AJS of the deviated side was significantly larger than the contralateral side (*P* < 0.045, Table 3). Moreover, the distance deviation of the condyle in the 3D coordinate system (X, Y, and Z) was significantly different in terms of X-value (*P* < 0.007), in which the value was smaller on the deviated side than the contralateral side. Furthermore, the Z value was significantly different (*P* < 0.001), which the value was higher on the deviated side than the contralateral side. However, the Y value was not significant (Table 3).

**Table 3:** Pre-treatment comparisons of temporomandibular joint spaces, and condylar position in the three coordinates

Measurements (mm)	Contralateral side	Deviated side	*P* value
Temporomandibular Joint Spaces			
SJS	2.58 ± 0.74	2.49 ± 0.88	0.393
MJS	2.78 ± 0.84	2.70 ± 0.92	0.690
AJS	1.87 ± 0.81	2.35 ± 1.01	0.045*
PJS	1.60 ± 0.68	1.25 ± 0.79	0.119
LJS	2.20 ± 0.88	2.35 ± 1.10	0.464
Anatomical landmarks (CO), Coordinate (mm)			
X	51.48 ± 3.16	49.94 ± 2.16	0.007**
Y	26.20 ± 8.42	25.51 ± 8.47	0.198
Z	69.97 ± 3.71	71.66 ± 3.53	0.001**

*P < 0.05, **P < 0.01

Table 4 shows the treatment effect in which the AJS decreased in both the deviated and contralateral sides post-treatment, but it was not significantly different. The SJS and PJS of the deviated side increased significantly (*P* < 0.004 and 0.006, respectively). In addition, the SJS and PJS of the contralateral side tended to increase, but no significant difference was observed. Pearson correlation showed negative correlation between the mean chin deviation and the superior (− 0.2) and posterior joint spaces (− 0.223) of the deviated side. Furthermore, the value of the comparison differences of the condylar position changes from the bilateral condyle head position to the X and Z axes decreased significantly (*P* < 0.029 and 0.023, respectively), while the value in the Y axis difference increased, although the difference was not significant.

**Table 4:** Pre- and post-treatment comparisons of temporomandibular joint spaces, condylar position and mandibular angles

	Measurements	Pre treatment	Post treatment	*P* value
Contralateral side	SJS	2.58 ± 0.74	2.88 ± 0.98	0.121
	MJS	2.78 ± 0.84	2.91 ± 1.04	0.233
	AJS	1.87 ± 0.81	1.84 ± 0.93	0.820
	PJS	1.60 ± 0.68	1.77 ± 0.84	0.316
	LJS	2.20 ± 0.88	2.35 ± 1.29	0.873
Deviated side	SJS	2.49 ± 0.88	2.98 ± 1.02	0.004*
	MJS	2.70 ± 0.92	2.75 ± 0.96	0.855
	AJS	2.35 ± 1.01	2.22 ± 0.99	0.119
	PJS	1.25 ± 0.79	1.86 ± 0.72	0.006*
	LJS	2.35 ± 1.10	2.50 ± 1.33	0.501
Anatomical landmarks Difference (mm)				
Co	|ΔX|	2.50 ± 1.56	1.64 ± 1.58	0.029*
	|ΔY|	2.12 ± 1.93	2.40 ± 1.99	0.133
	|ΔZ|	2.30 ± 1.57	1.82 ± 1.11	0.023*
Mandibular Angles				
Inclination Angle	FMSP-MMP	3.58 ± 2.02	3.17 ± 1.60	0.024*
	MP-HP	25.19 ± 6.38	24.99 ± 6.08	0.847
	HP-MMP	87.47 ± 2.15	87.56 ± 1.89	0.639

*P < 0.05 Considered significant

The mandible changes in pre- and post-treatment showed that the mandibular plane angle inclination decreased significantly after treatment (*P* < 0.024, Table 4).

## Discussion

TMD is considered the most prevalent non-dental orofacial pain condition and one of the most common oral and maxillofacial disorders [32]. The advanced condition of disc displacement in patients with TMD is observed more frequently on the deviated side of facial asymmetry [33]. Moreover, the severity of disc displacement is associated with the degree of mandibular asymmetry [34]. In addition, patients with menton deviation may increase the risk of disc displacement [35]. SS is one of the most frequently used treatments for individuals with TMD [18, 36]. TMD and mandibular deviation are related [5, 35], but whether SS can improve the mandibular deviation has not reported in patients with TMD. Thus, the main purpose of this study was to investigate a 3D detailed evaluation of the TMJ spaces, mandibular condyle, and the mandibular mediolateral positions following SS therapy in TMD patients with mandibular deviation willing to aid in clinical diagnosis and treatment.

The results of the present study show that the proportion of patients with ≥ 6 mm deviation decreased from 23.08% to 11.54% compared with pre-treatment, indicating that SS treatment can improve patients’ CD. This good improvement in correction of functional deviation indicated that it is preferable to check the possible causes of deviation to differentiate between the functional and skeletal ones before deciding to refer the case for orthognathic surgical treatment. Almost two-thirds of the treated patients showed improved chin deviation (69.23%). Approximately 30.77% of patients’ deviation was not improved, possibly because of the advanced condition of disc displacement and the deformity that is associated with the deviated side of mandibular asymmetry [34, 35].

In the present study, the AJS before treatment was significantly greater than the contralateral side. Both of them decreased post-treatment, thus supporting the results of the CBCT study of Ramachandran et al. [37] Hasegawa et al. [38] also reported that the condyle was displaced anteriorly and inferiorly following SS therapy. Therefore, SS treatment can effectively adjust the difference of bilateral joint space. Liu et al. [39] suggested the use of a splint that positioned the mandible anteriorly to maintain the disk in a normal relationship to the condyle. However, the posterior joint space was lesser in the deviated side than the contralateral side pre-treatment [40], thus increasing post-treatment requirements. A forward and downward movement to a myocentric position was suggested, similar to the findings of Ramachandran et al. [37], and this result demonstrates the findings of Hasegawa et al. [38].

The inconsistencies in anterior and posterior joint spaces in the bilateral joints indicate that before treatment, the position of the condyle in the deviated side was in a backward position [40]. However, no significant difference was observed in the bilateral joint space following therapy, indicating that SS can adjust the varying of the bilateral joint space and that the deviated condyle side moved anteriorly and inferiorly [38]. Similarly, the superior bilateral joint space increased post-treatment as the condyle moved downward [38], and the mandible also moved downward, increased the superior joint space and relieved the pressure between the tissues in the joint area.

Crawford et al. [41] reported a high correlation between the signs and symptoms of TMD and unfavorable condylar position determined by the occlusion with the help of the Panadent Condyle Position Indicator (CPI) value. In the present study, the 3D coordinate difference of the bilateral condyles was used for the statistical analysis of the condylar position changes pre- and post-treatment with SS. According to Ackermann et al. [42], the condyle records movement in 3D space as translation (forward/backward, up/down, right/left), combined with rotation on three perpendicular axes (yaw, pitch, and roll) [i.e., rotational displacements describe the coronal (roll), axial (yaw), and sagittal (pitch) planes of space].

In this study, the condylar position of the deviated side in the pre-treatment was in a posteromedial position, while the contralateral side was in an anterolateral position. Post-treatment, the value of the difference from the bilateral condyle head position to the X and Z axes decreased significantly. Therefore, it indicates that the bilateral condyles moved in a translational displacement (anteroposteriorly and mediolaterally). The results also show that the mandible rotated around the Z (roll) and Y axis (yaw) to the menton-deviation side [27], which made the position of bilateral condyles more coordinated. Xie et al. [42] demonstrated that the more the disc is displaced and deformed, the more the condylar height is shortened, and the mandible deviates. Their finding justifies the result of this study considering that when the mandibular deviation improved, this mostly resulted to release the pressure applied on the disc due to the post-treatment increase in the superior joint space and give more time for the disc to heal and return to its nearly previous position which almost minimizing the TMD symptoms.

The SS effect on the mandibular position decreased significantly post-treatment in the mandibular rotation angle (FMSP-MMP); after SS treatment, the mandible rotates around the midsagittal plane. The mandible rotated around the Z-axis (roll) post-treatment, making the mandible position move more to the middle of the face. This finding was obtained by Okeson [22], in which after treatment with TMD and returning the joint spaces and condyle to their stable ideal position, the mandible rotated to its standard position and through that, the MD was improved after SS therapy.

The limitations of this study included a small sample size, making the treatment effect based on the TMD categorization more difficult, the lack of precise evaluation of the changes in disc position that is only valid with the use of magnetic resonance imaging and the short-term follow-up period of the treatment effect.

## Conclusion

- As a non-surgical method, stabilization splint (SS) treatment can improve the facial asymmetry of patients with TMD and MD to a certain extent and recommended for functional mandibular deviation cases.
- SS treatment can improve the coordination of bilateral joint space and the condylar position and makes a relative movement of bilateral condylar positions in the X and Z axis, thus promoting the rotational movement around the Z (roll) and Y axis (yaw).
- SS can improve the mandible position and make the mandible rotate around the Z-axis (roll), making it more centered to the middle of the face. Accordingly, the facial asymmetry of TMD patients is improved.

## Data Availability

The datasets used and analyzed during the current study are available from the corresponding author on reasonable request.

## Abbreviations

TMD: Temporomandibular joint disorders
MD: Mandibular deviation
SS: Stabilization splints
CBCT: Cone Beam Computed Tomography
3D: Three dimensions
